# Exploring the association between parental rearing styles and medical students’ critical thinking disposition in China

**DOI:** 10.1186/s12909-015-0367-5

**Published:** 2015-05-14

**Authors:** Lei Huang, Zhaoxin Wang, Yuhong Yao, Chang Shan, Haojie Wang, Mengyi Zhu, Yuan Lu, Pengfei Sun, Xudong Zhao

**Affiliations:** 1 Tongji Hospital, Tongji University School of Medicine, Shanghai, China; 2 Yangpu Hospital, Tongji University School of Medicine, Shanghai, China; 3 Tongji University School of Medicine, Shanghai, China; 4 Department of Psychosomatic Medicine, Shanghai East Hospital Affiliated toTongji University, Shanghai, China; 5 The Psychological Counseling Center, Tongji University, Shanghai, China

**Keywords:** Parental rearing styles, Critical thinking disposition, Medical students

## Abstract

**Background:**

Critical thinking is an essential ability for medical students. However, the relationship between parental rearing styles and medical students’ critical thinking disposition has rarely been considered. The aim of this study was to investigate whether parental rearing styles were significant predictors of critical thinking disposition among Chinese medical students.

**Methods:**

1,075 medical students from the first year to the fifth year attending one of three medical schools in China were recruited via multistage stratified cluster sampling. The Chinese Critical Thinking Disposition Inventory(CTDI-CV) and The Egna Minnen av Barndoms Uppfostran (EMBU) questionnaire were applied to collect data and to conduct descriptive analysis. Stepwise multiple linear regression was used to analyze the data.

**Results:**

The critical thinking disposition average mean score was 287.44 with 632 participants (58.79%) demonstrating positive critical thinking disposition. Stepwise multiple linear regression analysis revealed that the rearing styles of fathers, including “overprotection”, “emotional warmth and understanding”, “rejection” and “over-interference” were significant predictors of medical students’ critical thinking disposition that explained 79.0% of the variance in critical thinking ability. Rearing styles of mothers including “emotional warmth and understanding”, “punishing” and “rejection” were also found to be significant predictors, and explained 77.0% of the variance.

**Conclusions:**

Meaningful association has been evidenced between parental rearing styles and Chinese medical students’ critical thinking disposition. Parental rearing styles should be considered as one of the many potential determinant factors that contribute to the cultivation of medical students’ critical thinking capability. Positive parental rearing styles should be encouraged in the cultivation of children’s critical thinking skills.

## Background

It is generally agreed upon that one of the main goals of education, at any level, is to help students develop general thinking skills, particularly those pertaining to critical-thinking [1]. Critical thinking is a valuable skill that is necessary to effectively cope with mental and spiritual matters as well as to evaluate people, policies and institutions, thereby facilitating the avoidance of social difficulties. Two thousand years ago, Aristotle had already realized the importance of critical thinking in education [2]. Since 1980, critical thinking has been an avowed goal in higher education and has been widely considered an essential quality of a citizen in a democracy [3]. Many countries have sought to cultivate critical thinking in their students. In 1985, the Association of American Colleges pointed out that all students should learn “to reason well, to recognize when reason and evidence are not enough, to discover the legitimacy of intuition, to subject data to the probing analysis of the mind”, which emphasized the importance of critical thinking [4].

As the importance of critical thinking has become widely recognized and research on the subject has grown increasingly popular, an increased amount of attention has also been allocated to the role of this skill in the healthcare profession. In 2002, “Critical thinking and research”was defined by the Institute for International Medical Education (IIME) in the Global Minimum Essential Requirements (GMER) as an essential competency that a medical graduate should possess [5]. Practically speaking, life is precious and medicine is a rigorous and challenging discipline. Doctors have a tense and busy work schedule, and are often faced with uncertainties when dealing with a variety of clinical problems such as rapid changes of disease. Therefore, the ability to make an independent, precise and quick decision and work toward resolving patient problems is extremely important for doctors. Critical thinking helps doctors evaluate existing knowledge, technology and information critically and make accurate judgments in clinical practice, so as to ensure that patients receive the best treatment possible [6-8]. Moreover, with the development of medicine and the big bang of medical knowledge, critical thinking encourages doctors to challenge apparent certainties and uncover useful information. Previous studies have testified to the positive correlation between critical thinking and clinical competence [9-12], academic success [13,14] and research skills [15]. Therefore, it is particularly important to encourage medical students’ critical thinking skills, as evidence suggests that it may be highly beneficial to their future careers.

Critical thinking has been defined in many ways by scholars, with the following most popular definition stated in 1990 in the Delphi Report by the American Philosophical Association: critical thinking is a purposeful, self-regulatory judgment which results in interpretation, analysis, evaluation and inference, as well as explanation of the evidential conceptual, methodological, criteriological or contextual considerations upon which that judgment was based [16]. It is argued that critical thinking is both a learned collection of critical thinking skills and a disposition towards engaging in the reasoning process [17]. A significant correlation between critical thinking disposition and skills has been found in previous studies [18,19]. These studies suggest that the disposition and habits of a mind are crucial in the exercise of critical thinking, and describe the trend of critical thinking as an inner motivation to solve problems and make decisions by thinking. Therefore, to facilitate the development of critical thinking skills, there is a need to foster critical thinking disposition [20].

Although the importance of critical thinking in medical students is well recognized, most studies have focused on aspects of medical education, such as educational experience [21], teaching method [22] and learning style [23]. Undoubtedly, education plays a significant role in nourishing medical students’ critical thinking. However, critical thinking is not only related to education, but is also related to personality factors [24-26], social environment and culture [27,28]. The family environment is recognized as one of the proximal environments where socialization and development take place. Among various factors related to the growth of children in a family, parental rearing styles, the combination of parental behaviors which appear regularly and can lead to enduring parenting habits [29,30] may have a vital influence on children’s development including their critical thinking disposition.

The term “parental rearing style” was first formulated in 1959 by Schaefer, who proposed four parental styles: authoritative (high warmth and low control), overprotective (high warmth and high control), authoritarian (affective coldness and high control) and neglecting (affective coldness and low control) [31]. Following this line of study, Diana Baumrind developed a typology composed of three parenting types: authoritative (high in both demandingness and responsiveness), authoritarian (high in demandingness but low in responsiveness) and permissive (high in responsiveness and low in demandingness). This typology set the research agenda for the field of parenting styles [32], Based on the four-step classification developed by Maccoby and Martin [33], Baumrind subsequently added a fourth type, the neglectful parenting style (low in both responsiveness and demandingness [34]). Research has shown that parental rearing styles reflect prevailing cultural values. Typically, mothers from Western countries promote individualistic and independent behaviour in their children by using reasoned control, openly expressing their warmth and intimacy, and frequently praising their children. In contrast, Chinese culture places more emphasis on social hierarchy and discipline, and Chinese parents are more likely to display relatively higher rates of the authoritarian rearing style.

Parental rearing styles have a significant impact on children’s personality formation. Inappropriate rearing styles may lead to personality disorders, as parental care, freedom control and autonomy denial are important factors in the development of personality disorders [35]. On the other hand, evidence for a relationship between critical thinking disposition and personality has also been found. The personality traits of openness to experience [24], thinking introversion, response bias, altruism, autonomy, complexity, theoretical orientation [25] and disposition of concern for truth [26] are significantly correlated with critical thinking. As a result, we formed the hypothesis that personality may be the mediating factor in the association between parental rearing styles and students’ critical thinking disposition. To our knowledge, there is only one study investigating the relationship between perceived parenting style and critical thinking that presented a clear relation between rearing styles and critical thinking skills with cognitive learning styles [36]. However, there is still an absence of empirical studies examining the association between parental rearing styles and critical thinking among medical students. Due to the importance of critical thinking for medical students and the importance of parenting style in the developmental process, it is both reasonable and intriguing to propose this study to bridge this gap.

## Methods

### Participants

In this descriptive, cross-sectional study, we recruited 1,241 medical students from the first year to the fifth year attending one of three universities: Tongji University School of Medicine (Shanghai), Medical College of Soochow University (Jiangsu province) and Gannan Medical University (Jiangxi province) via multistage stratified method. These universities are classed as first rank institutions in Eastern China. After graduating from senior high school, undergraduate students must attend medical school for five years in order to receive their bachelor degree in medicine, and this period is regarded as the most important stage in medical education. For better representativeness, our sampling was stratified by number of years of school attended among undergraduate medical students. Approximately 80–100 students were randomly selected from each year from each of the three universities.

### Instruments and procedures

The Chinese Critical Thinking Disposition Inventory (CTDI-CV) was used to assess medical students’ critical thinking disposition. This questionnaire was modified by Peng [37] according to Facione’s California Critical Thinking Dispositions Inventory (CCTDI ) [38] and has been widely implemented in China [39,40], especially in the field of research involving medical students. The CTDI-CV is a standardized, 70-item, multiple-choice test that can be divided into seven dimensions: truth seeking, open-mindedness, critical thinking, self-confidence, inquisitiveness and cognitive maturity, analyticity and systematicity. The overall content validity index (CVI) was 0.89, with subscale CVIs ranging from 0.6 to 1. The overall alpha value was 0.90 and the subscale alpha values ranged from 0.54 to 0.77. A total score of 280 or above and subscale scores above 40 were considered to indicate positive critical thinking disposition.

The Egna Minnen av Barndoms Uppfostran (EMBU) questionnaire was used to assess participants’ own memories of parental rearing behavior. This questionnaire is comprised of 81 questions grouped into 15 subscales and 2 additional questions referring to the consistency and strictness of parental rearing behavior [41]. The Chinese version of the questionnaire was revised by Yue [42] using principal component factor analysis. Cultural differences between Chinese individuals and Western individuals are considered in the revised version, which consists of 115 items and 11 subscales, with 58 items comprising 6 subscales (emotional warmth and understanding, punishing, over-interference, favoring subjects, rejection and overprotection) related to the father, and 57 items comprising 5 subscales (emotional warmth and understanding, over-interference and overprotection, rejection, punishing and favoring subjects) related to the mother. This inventory has been widely used in China [43-45]. Responses are rated on a 4-point scale Likert scale. The test-retest reliability is between 0.58 and 0.82. The split-half reliability is between 0.50 and 0.91, and the internal consistency reliability is between 0.59 and 0.88.

Demographic data such as gender, age, grade and program were collected using a separate questionnaire created by our team.

Data was collected at the end of the academic semester from medical students at each of the three medical schools. Prior to data collection, the purpose of the study and participants’ rights as subjects in research were explained to all students. Informed consent was obtained. Three research fellows from our team supervised the investigation procedure and data input.

### Statistical analyses

Descriptive analysis and stepwise multiple linear regression were used to analyze the data using SPSS version 19.0. A significant p-value <0.05 was found.

### Ethical considerations

Ethics clearance for the project was obtained from the Ethics Review Committee of Tongji Hospital and Tongji University (Registration Number K-2014-020). The purpose of the study was explained to participants and participation was voluntary.

## Results

### Characteristics of participants

A total of 1,241 medical students were recruited of which 166 were excluded due to incomplete data, corresponding to a response rate of 86.62%. 358 (33.30%) students were attending Gannan Medical University, 329 (30.60%) students were attending the Medical College of Soochow University and 388 (36.10%) students were attending Tongji University School of Medicine. The sample consisted of 522(48.56%) male and 553 (51.44%) female participants. Participant age ranged from 18–27 years (mean =22.06, SD = 1.76) with the majority of participants falling between 20 and 24 years of age (n = 979, 85.49%). 556 (51.72%) students were the only child in family while 519 (48.27%) students had siblings. At the time of data collection 248 (23.07%) students were in the first year, 198 (18.42%) students were in the second year, 244 (22.70%) students were in the third year, 222 (20.65%) students were in the fourth year and 163 (15.16%) students were interns (Table 1).

**Table 1:** **Characteristics of participants**

Demographic	n	%
**School**		
Gannan Medical University	358	33.30
Medical College of Soochow University	329	30.60
Tongji university School of Medicine	388	36.10
**Gender**		
Male	522	48.56
Female	553	51.44
**Age**		
18-19	71	6.60
20-24	919	85.49
25-27	85	7.90
**Only child or not**		
Yes	556	51.72
No	519	48.27
**Year**		
First year	248	23.07
Second year	198	18.42
Third year	244	22.70
Fourth year	222	20.65
Fifth year	163	15.16

### Descriptive analysis of CTDI-CV and EMBU

Scores on the CTDI-CV ranged from 216–383 with a mean score of 287.44 (SD = 29.08). The index score was 280, indicating that the critical thinking disposition of Chinese medical students was at a positive level. 632 (58.79%) participants had a total score above 280, which indicates a positive attitude towards critical thinking while 443 (41.21%) participants scored below 280. It is difficult to compare the rearing styles of fathers with that of the mothers because the subscales and number of items are not designed in a one to one correspondence. Items in the “favoring subjects” subscale were only filled in by participants with at least one sibling. As the one-child policy has been in effect in China since 1979, many families have only one child, and most participants in our study were only children. As a result, 10 items in these two subscales were ignored in order to preserve the integrity of the data (Table 2 and Table 3).

**Table 2:** **Descriptive analysis of CTDI-CV**

Dimensions	Mean ± SD	Max	Min	Positive attitude n (%)	Negative attitude n (%)
Truth seeking	37.05 ± 6.07	56.00	18.00	713(66.32)	362(33.68)
Open-mindedness	41.06 ± 5.38	57.00	24.00	661(61.49)	414(38.51)
Analyticity	43.70 ± 5.47	60.00	27.00	819(76.19)	256(23.81)
Systematicity	38.86 ± 6.11	60.00	19.00	459(42.70)	616(57.30)
Self-confidence	41.40 ± 5.85	60.00	22.00	659(61.30)	416(38.70)
Inquisitiveness	44.68 ± 6.10	60.00	24.00	851(79.16)	224(20.84)
Cognitive maturity	40.69 ± 6.22	57.00	17.00	654(60.84)	421(39.16)
**Total score**	287.44 ± 29.08	383.00	216.00	632(58.79)	443(41.21)

**Table 3:** **Descriptive analysis of EMBU**

Dimensions	Items	Mean ± SD	Max	Min
**Father**				
Emotional warmth and understanding	19	53.22 ± 9.04	73.00	25.00
Punishing	12	16.59 ± 4.76	42.00	12.00
Over-interference	10	19.87 ± 3.50	34.00	11.00
Rejection	6	8.60 ± 2.71	21.00	6.00
Overprotection	6	10.50 ± 2.37	20.00	5.00
**Mother**				
Emotional warmth and understanding	19	57.40 ± 9.38	76.00	27.00
Over-interference and Overprotection	16	34.28 ± 5.90	55.00	20.00
Rejection	8	11.78 ± 3.57	28.00	8.00
Punishing	9	11.94 ± 3.90	33.00	9.00

### Association between parental rearing styles and medical students’ critical thinking disposition

In order to investigate the relationship between students’ critical thinking and the rearing styles of fathers, we took the sum of critical thinking scores as the dependent variable, and the five types of fathers rearing styles as the independent variables. To determine the main factors, we used linear regression and the stepwise method (Stepwise Criteria: Probability-of-F-to-enter < = .050, Probability-of-F-to-remove > = .100). Of the four models, the fourth had the highest R-squared value (0.792) and was consequently selected as the final model. Furthermore, we found that four of the fathers rearing styles (overprotection, emotional warmth and understanding, rejection, and over-interference) showed correlations with the CTDI-CV (p < 0.05). Moreover, “overprotection” and “rejection” showed justified negative correlations with the CTDI-CV, while “emotional warmth and understanding” and “over-interference” showed positive correlations (Table 4 and Table 5).

**Table 4:** **Stepwise model summary between critical thinking and the rearing styles of fathers & mothers**

Model		R	R square	Adjusted R square	Std. error of the estimate	Change statistics				
						R square change	F change	df1	df2	P
Fathers	1	0.784^a^	0.615	0.614	27.528	0.615	125.884	1	1073	0.000
	2	0.847^b^	0.717	0.715	26.848	0.717	56.087	1	1072	0.000
	3	0.865^c^	0.748	0.746	26.758	0.748	8.219	1	1071	0.004
	4	0.890^d^	0.792	0.79	26.705	0.792	5.274	1	1070	0.022
Mothers	1	0.838^A^	0.702	0.699	27.172	0.702	157.502	1	1073	0.000
	2	0.863^B^	0.745	0.742	26.508	0.745	55.482	1	1072	0.000
	3	0.879^C^	0.773	0.77	26.452	0.773	5.516	1	1071	0.019

^a^Predictors: (Constant), Overprotection.

^b^Predictors: (Constant), Overprotection, Emotional Warmth and Understanding.

^c^Predictors: (Constant), Overprotection, Emotional Warmth and Understanding, Rejection.

^d^Predictors: (Constant), Overprotection, Emotional Warmth and Understanding, Rejection, Over-interference.

^A^Predictors: (Constant), Emotional Warmth and Understanding.

^B^Predictors: (Constant), Emotional Warmth and Understanding, Punishing.

^C^Predictors: (Constant), Emotional Warmth and Understanding, Punishing, Rejection.

**Table 5:** **The results of regression between critical thinking and the rearing styles of fathers & mothers**

Model			Unstandardized coefficients		Standardized coefficients	t	P
			B	Std. error	Beta		
Fathers	4	**(Constant)**	274.742	6.97		39.419	0.000
		Overprotection	−2.591	0.382	−0.241	−6.791	0.000
		Emotional warmth and understanding	0.682	0.103	0.212	6.622	0.000
		Rejection	−1.374	0.4	−0.112	−3.438	0.001
		Over-interference	0.662	0.288	0.08	2.296	0.022
Mothers	3	**(Constant)**	267.083	7.532		35.46	0.000
		Emotional warmth and understanding	0.769	0.097	0.248	7.948	0.000
		Punishing	−1.18	0.317	−0.158	−3.721	0.001
		Rejection	−0.822	0.35	−0.101	−2.349	0.000

a. Dependent Variable: Sum of critical thinking scores.

Regarding the relationships between students’ critical thinking and the rearing styles of mothers, all are as same as reported in the above analysis except values obtained when the four types of mother’s rearing styles are used as independent variables. As a result, among the three models, the third has the highest R-squared value (0.773) and was selected as the final model. We subsequently found that the three mothers’ rearing styles (emotional warmth and understanding, punishing and rejection) showed correlations with the CTDI-CV (p < 0.05). Furthermore, “punishing” and “rejection” displayed negative correlations with the CTDI-CV, while “emotional warmth and understanding” showed positive correlations (Table 4 and Table 5).

## Discussion

The average mean score on the CTDI-CV was above 280, indicating that Chinese medical students have a positive attitude towards critical thinking. This finding is similar to results reported in domestic studies conducted by Zhang Ya-qing [39]and Chen Jin [46]. However, only 58.19% of the participants showed a positive attitude towards critical thinking, which is lower than the 78% of American undergraduates that displayed a positive attitude towards critical thinking in a study conducted by Giancarlo and Facione [47]. This comparison suggests that Chinese medical students’ critical thinking disposition is inferior to students from Western countries, a finding echoed in research conducted by Tiwari [48] and Yeh ML [49]. The differences in terms of critical thinking between China and Western countries may be due to cultural differences [48] and differences in the education systems [49]. Chinese culture does not always value critical questioning and unconventional views [50], and children who question authority are often considered impolite and criticized for their behavior. It is generally believed that students are not encouraged to ask teachers questions because teachers are afraid of losing face if they are unable to provide an answer for their students [51].

Family is not only an important cultural carrier, but is also the first learning setting children are exposed to. Parental rearing style is considered a specific educational medium through which Chinese culture and social values are passed on to children. In this study, results showed that several patterns of parental rearing style are significant in predicting students’ critical thinking, which emphasizes the importance of rearing behavior in a family. This result is consistent with a study conducted in Iran, which found that children whose parents behave with authority display higher levels of adaption, psychological maturity, psycho-social efficiency, self-confidence and educational success, while children of authoritarian parents exhibit weak social skills and low self-confidence [36].

China has experienced radical changes due to modernization and globalization since 1979, the same year in which the one-child policy was implemented. As a result, parental rearing styles have most likely been adjusted. Studies support the notion that the implementation of the one-child policy has lead to “child- centered” rearing practices in contemporary Mainland China. Nowadays, Chinese parenting practices appear to be both authoritarian and authoritative in styles [52]. The Chinese term “guan”, which means to “govern”, as well as to love may best describe this parental rearing style [53].

Our findings showed that a parental rearing style consisting of “emotional warmth and understanding” is a positive predictor of medical students’ critical thinking disposition, indicating that “warmth and understanding” should be encouraged in parent–child interactions in order to facilitate critical thinking in the child. Due to the one-child policy, many children in China do not have siblings. Chinese parents are generally immensely devoted to their only child and often make significant sacrifices to meet their child’s needs [54]. Far more attention has been paid to children of this generation in comparison to previous generations. Also, more and more parents in mainland China are highly-educated, and Chinese mothers with a higher education level are more inclined to adopt authoritative rearing styles [55]. This indicates that well-educated parents may have a better understanding of the rearing styles practiced in Western culture, which value the explicit emotional expression that is beneficial to children’s critical thinking cultivation over subtle or implicit expression of emotion.

As a result of the impact that traditional Chinese culture and the education system have on parents, a hierarchy still exists between parents and children in most Chinese families. Parents who strongly adhere to traditional values are subsequently more likely to view themselves as teachers of their children. In this kind of family environment children are expected to be obedient and respectful to their elders, such as teacher and parents. If a child’s behavior goes against the will of his or her parents, rejection or punishment, consistent with an authoritarian parenting style, may occur. The present study revealed that parental rearing styles involving rejection and mothers’ rearing styles involving punishment are negative predictors of medical students’ critical thinking disposition.

Studies have found differences in parenting between mothers and fathers. One such study found mothers to be highly responsive but relatively undemanding, whereas fathers were found to be highly demanding but frequently coercive and unresponsive [34]. In our study, fathers’ “overprotection” and mothers’ “punishing” were negative predictors. However, mothers’ “overprotection and over-interference” and fathers’ “punishing” were not significant in predicting students’ critical thinking disposition, which indicates that high warmth and high control by fathers as well psychological [mental] and bodily punishment by mothers has a negative effect on students’ critical thinking disposition. It also suggests that the typical image of Chinese parents as a strict father and a gentle mother is good for children’s critical thinking development. Contrary to our expectations, fathers’ “over-interference” was significantly positive for predicting students’ critical thinking disposition. We found that an authoritarian parental rearing style is not always detrimental to child development [56]; in Chinese culture the parenting style of “guan” is characterized by care and warmth rather than control [57]. Moreover, fathers are more concerned about their children’s studies while mothers are concerned about their daily lives. As a result, the father’s “over-interference” may promote the child’s development, including the development of their thinking style.

### Strength and limitation

This study and its design have several strengths and limitations. To our knowledge, this is the first study examining the relationship between critical thinking disposition and parental rearing styles among medical students. Our sample is sizable and representative. However, a limitation of the study is that all data are based on self-report questionnaires and parental rearing styles are based solely on participant memory. While these instruments have been validated for the Chinese population, the use of self- report assessment and retrospective study may reduce the validity of the data. As this study is cross-sectional and descriptive, in order to provide more compelling evidence concerning the effects of parenting styles on students’ critical thinking disposition, a longitudinal and prospective study is suggested for future research.

## Conclusions

Critical thinking is a particularly important disposition for medical students and for their future careers as doctors. There is an association between certain parental rearing styles and Chinese medical students’ critical thinking disposition. Parental rearing styles should be considered as one of the many potential determinant factors that contribute to the cultivation of medical students’ critical thinking capability. “Emotional warmth and understanding” is a positive rearing style and should be encouraged, while “rejection” is a negative style and should be abandoned in order to facilitate students’ development of critical thinking. Evidence indicates that parents should pay more attention to their rearing styles in order to promote children’s critical thinking disposition. While more research is needed in this area, our findings indicate that parental rearing styles must be taken into consideration in the cultivation of medical students’ critical thinking skills.
